# Dry powder inhaled compound delivery for early pre-clinical *in vivo* efficacy studies

**DOI:** 10.1186/1476-9255-10-S1-P36

**Published:** 2013-08-14

**Authors:** Robert Ives, Lindsay Butterfield, John Farr, Shaun Bater, Tony Nials, Lewis Entwistle, Doug Ball, Edith Hessel

**Affiliations:** 1 GlaxoSmithKline, Stevenage, UK

## Introduction

Inhaled dry powders are the preferred route of delivery for many respiratory medicines. Toxicology studies are routinely carried out on inhaled dry powders whereas early pre-clinical *in vivo* efficacy studies often use aqueous formulations. Ideally evaluation of in- vivo efficacy, duration of action and DMPK of novel compounds, delivered as dry powders, should be carried out at an earlier stage in the drug development process. This would allow direct comparisons to be made with toxicology data to establish a therapeutic index and enable a more relevant prediction of clinical efficacy.

## Methods

We have evaluated the efficacy of Fluticasone Propionate delivered as a dry powder, using an Aerosolised Dust Generation (ADG) tower and a mini-Wrights Dust Feeder II (WDF), in a rat model of LPS induced pulmonary neutrophilia. To more accurately control the doses the animals received we used an Aerosol Particle Sizer (APS) to measure, in real time, the airstream compound concentration. Using this inhaled delivery system allows a more physiologically relevant delivery and distribution to the lungs. The ADG tower and WDF were identical to those used in house for standard safety assessment studies with novel respiratory target molecules.

## Results

We have shown a dose-related inhibition of LPS-induced pulmonary neutrophilia following inhaled delivery of a range of doses of Fluticasone Priopionate. Analysis of lung and plasma concentrations of Fluticasone, plus measurement of airstream compound concentration enabled more accurate dosimetry calculations.

## Conclusion

Use of this standard inhaled dry powder delivery technique may more closely link early pre-clinical efficacy and toxicology studies and allow direct comparisons to be made to establish a therapeutic index, and therefore potentially a more relevant prediction of clinical efficacy.

